# Sensing While Drilling and Intelligent Monitoring Technology: Research Progress and Application Prospects

**DOI:** 10.3390/s25206368

**Published:** 2025-10-15

**Authors:** Xiaoyu Li, Zongwei Yao, Tao Zhang, Zhiyong Chang

**Affiliations:** 1 College of Biological and Agricultural Engineering, Jilin University, Changchun 130022, China; lixy1516@163.com; 2 Key Laboratory of Bionic Engineering, Ministry of Education, Jilin University, Changchun 130022, China; 3 School of Mechanical and Aerospace Engineering, Jilin University, Changchun 130000, China; yzw@jlu.edu.cn; 4 College of Communication Engineering, Jilin University, Changchun 130000, China; 5 School of Engineering and Technology, China University of Geosciences, Beijing 100083, China

**Keywords:** sensing while drilling, intelligent monitoring, petroleum drilling, research progress, application prospects

## Abstract

Obtaining accurate information on stratigraphic conditions and drilling status is necessary to ensure the safety of the drilling process and to guarantee the production of oil and gas. Sensing while drilling and intelligent monitoring technology, which employ multiple sensors and involve the use of intelligent algorithms, can be used to collect downhole information in situ to ensure safe, reliable, and efficient drilling and mining operations. These approaches are characterized by effective sensing and comprehensive utilization of drilling information through the integration of multi-sensor signals and intelligent algorithms, a core component of machine learning. The article summarizes the current research status of domestic and international sensing while drilling and intelligent monitoring technology using systematically collected relevant information. Specifically, first, the drilling-sensing methods used for in situ acquisition of downhole information, including fiber-optic sensing, electronic-nose sensing, drilling engineering-parameter sensing, drilling mud-parameter sensing, drilling acoustic logging, drilling electromagnetic wave logging, and drilling seismic logging, are described. Next, the basic composition and development direction of each sensing technology are analyzed. Subsequently, the application of intelligent monitoring technology based on machine learning in various aspects of drilling- and mining-status identification, including bit wear monitoring, stuck drill real-time monitoring, well surge real-time monitoring, and real-time monitoring of oil and gas output, is introduced. Finally, the potential applications of sensing while drilling and intelligent monitoring technology in deep-earth, deep-sea, and deep-space contexts are discussed, and the challenges, constraints, and development trends are summarized.

## 1. Introduction

Energy security is crucial for ensuring the sustainable development of national and regional economies. Currently, fossil fuels, primarily oil and natural gas, dominate global energy consumption, and the demand for these resources is expected to continue growing for the foreseeable future. Since the 1930s, 25 oil and gas basins have been successfully developed worldwide, and these contribute to 35% of global oil and gas production. However, due to limitations in exploration and development capabilities, over 90% of tight oil and gas reserves and 65% to 90% of shale oil and gas resources remain untapped [1]. These resources represent significant potential reserves, and their exploration and development are of great value in promoting national development and improving public welfare.

Drilling engineering is a critical component of oil and gas exploration and development. Drilling into potentially oil- and gas-bearing formations is the most direct method for locating these resources. However, drilling is a costly and inherently risky endeavor. To achieve economical, safe, and efficient oil and gas extraction, two key factors are essential: first, the accurate acquisition of complex formation and drilling data, which then serve as the foundation for drilling operations and resource assessment [2]; and second, the integration and utilization of formation and drilling data [3], which ensures efficient resource extraction and mitigates drilling risks. In traditional drilling processes, data collection primarily occurs at the surface, relying on monitoring of drilling-rig conditions and analysis of returned drilling fluids to assess downhole conditions [4]. This approach heavily depends on the experience of on-site technicians and, because it is not carried out in situ, inevitably leads to data gaps and delays, introducing significant uncertainties and potential risks that hinder the overall progress of oil and gas exploration.

With the rapid advancement of sensor technology and artificial intelligence, the introduction of in situ monitoring and intelligent algorithms has made real-time drilling sensing and intelligent monitoring possible, offering new methods for achieving efficient and safe oil and gas exploration [5]. Real-time drilling sensing and intelligent monitoring utilize sensors for detection of temperature, pressure, and hydrocarbons to collect multi-modal downhole data in real time. These data are transmitted to the surface via channels such as fiber optics, effectively addressing issues of data loss and delay. Additionally, leveraging intelligent algorithms with capacities such as big data analytics and pattern recognition, the system enables intelligent monitoring of drilling conditions through the modeling and analysis of multi-modal downhole data. This technology has been applied in areas such as oil and gas production forecasting, drill-bit wear and stuck-pipe monitoring, and kick detection [6].

Real-time drilling sensing and intelligent monitoring rely on two key processes: downhole data acquisition and state-recognition monitoring. Fundamentally, downhole data acquisition involves the use of various sensors to measure parameters in multiple phases (gas, liquid, and solid) to reflect actual formation conditions and drilling-rig status. State-recognition monitoring, on the other hand, employs machine learning and artificial intelligence algorithms to classify patterns and perform regression analysis on downhole data, enabling the detection of drilling states and operational conditions. Therefore, the first two sections of this paper review downhole data acquisition and state-recognition monitoring, respectively, introducing existing downhole sensing methods and discussing the application of intelligent algorithms in downhole state recognition. Building on this foundation, the paper analyzes the potential applications of intelligent monitoring technology in deep-earth, deep-sea, and deep-space exploration, proposing feasible strategies for implementation. Finally, summarize the future development trends of downhole sensing and intelligent monitoring technologies.

## 2. In Situ Downhole Information Acquisition Technology While Drilling

**Figure 1:**
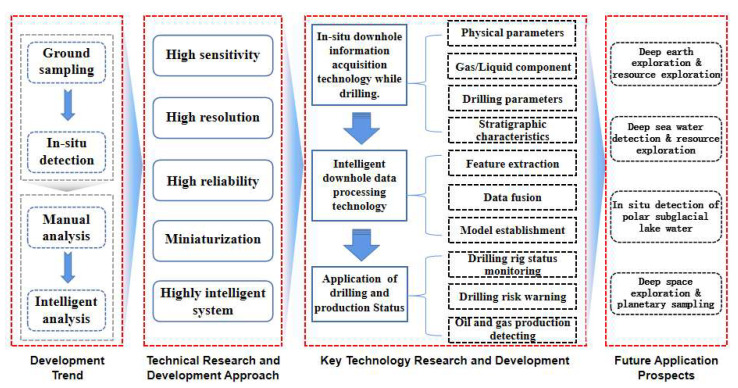
Conceptual framework for research on technology for intelligent monitoring while drilling.

The technology of downhole sensing and intelligent monitoring aims to address issues such as high latency and poor detection accuracy that are associated with surface sampling and manual analysis methods. Therefore, the research approach starts with downhole in situ data-acquisition methods centered around downhole sensing, exploring the implementation and characteristics of downhole in situ detection. Subsequently, it investigates the advantages of intelligent data-processing technologies, which are centered on intelligent algorithms, over manual analysis. Ultimately, the integration of these two aspects results in intelligent recognition and monitoring of drilling and production status, as illustrated in Figure 1.

Real-time, reliable, and comprehensive acquisition of downhole information is a prerequisite for making accurate judgments about the real-time drilling and production status. This primarily includes the detection of downhole physical parameters and gas–liquid composition, the identification of formations encountered during drilling, and monitoring of drilling parameters. Monitoring of downhole temperature, pressure, and gas–liquid composition is required throughout the entire drilling process. The distribution of the wellbore temperature field and pressure changes directly affect the performance of drilling fluids and the pressure balance within the wellbore. Meanwhile, temperature and pressure changes in the near-bit area, along with gas–liquid composition, are crucial variables in analyses of the characteristics of the formations encountered and evaluations of hydrocarbon production. On this basis, by integrating engineering drilling parameters, mud-parameter monitoring, and real-time formation data, multidimensional information on logging while drilling (LWD) can be effectively obtained. Currently, the methods of information acquisition can be categorized into surface acquisition and downhole in situ acquisition. Compared to surface-based acquisition methods, which involve monitoring drilling return fluids, downhole in situ acquisition while drilling can capture multimodal signals throughout the drilling process using various sensors, thereby more comprehensively reflecting data on formations at different downhole locations. This significantly enhances the timeliness and accuracy of the information.

### 2.1. Fiber-Optic Temperature Sensing

The distribution of wellbore and formation temperatures significantly impacts wellbore pressure control, drilling fluid rheology, and wellbore stability, as well as the thermal-fatigue failure and corrosion rate of drilling tools [7]. Therefore, it is essential to collect real-time temperature data from the formation during the drilling process to accurately assess the thermal conditions to which the equipment is subjected, adjust drilling parameters in a timely manner, and enhance drilling efficiency. The sensible heat increment [8] of the formation temperature can be expressed as follows:

(1) $$Q_{s}=mc_{p}(T_{e}-T_{0})$$

In Equation (1), *Q_s_* represents the sensible heat accumulation; m denotes the mass; *c_p_* is the constant specific heat; *T_e_* is the wellbore temperature; and *T*_0_ is the initial temperature of the device. Therefore, when assessing the heat exposure of electronic devices, the key lies in monitoring the wellbore temperature *T_e_*. Accurately detecting the real-time temperature within the wellbore can help field operators to promptly assedd the sensible heat increment and operational status of downhole equipment in the formation, ensuring the reliable progress of drilling operations. Extensive literature research and downhole field experiments have demonstrated that fiber-optic temperature-sensing technology is an effective method for real-time temperature monitoring during drilling [7,9].

#### 2.1.1. Fiber Bragg Grating Temperature Sensing

Fiber Bragg grating (FBG) can be utilized for downhole temperature sensing at fixed points. With the grating embedded at specific locations along the optical fiber, temperature measurements are conducted and the temperature signals rapidly transmitted back to the surface via the optical fiber, providing essential temperature data to support drilling operations. The fundamental principle of FBG is as follows: The grating is inscribed into the optical fiber using a high-intensity infrared femtosecond laser or an ultraviolet laser phase mask, thereby creating a periodic variation in the refractive index within the fiber core. This periodic variation interferes with light of a specific wavelength known as the Bragg wavelength [10].

(2) $$\lambda_{b}=2\cdot n_{eff}\cdot \Lambda$$

In Equation (2), *λ_b_* represents the Bragg wavelength; Λ denotes the grating period; *n_eff_* is the effective refractive index of the fiber core. Changes in ambient temperature affect the linear thermal expansion and the thermo-optic effect (TOE) of the fiber, thereby altering the effective period of the grating [10].

(3) $$\frac{\partial \lambda }{\partial T}=2\cdot \;\left(\Lambda \frac{\partial n_{eff}}{\partial T}+n_{eff}\frac{\partial \Lambda }{\partial T}\right)$$

The temperature changes within the wellbore are thus converted into alterations in optical signals, enabling temperature measurement. The advantage of FBGs lies in their high sensitivity to temperature variations; moreover, changes in the effective period offer higher signal resolution compared to peak changes, allowing for more precise temperature detection. However, the delicate surface structure of Bragg gratings cannot withstand significant thermal expansion, necessitating a focus on structural or material protection when gratings are applied in downhole environmental studies. At the same time, fiber Bragg gratings are often affected by the cross effects of temperature and pressure, namely the thermal-expansion effect caused by temperature and the axial tension and compression caused by pressure changes. Therefore, in practical applications, specific decoupling methods are usually needed to decouple the response differences between strain and temperature, or special packaging designs are used to offset the effects of either temperature or pressure [11].The FBG fiber temperature sensor designed by National Taiwan University has been successfully applied in a 1195-m-deep geothermal exploration well, where the sensor functioned normally under a formation pressure of 11.65 MPa and measured a maximum formation temperature of 75 °C [12]. In deeper drilling processes, formation temperatures can often reach several hundred degrees Celsius, requiring fibers with excellent thermal stability, a requirement that imposes stringent demands on the material selection for the fibers. Sapphire fiber Bragg gratings (SFBG), which are made from single-crystal sapphire fibers, have a high melting point of approximately 2045 °C, making them suitable as high-temperature measurement materials. Shenzhen University has developed an ultra-high-temperature sensor based on SFBG [13]. To protect the grating probe, the research team engraved the grating onto the tip of a single-crystal sapphire fiber using femtosecond laser technology and encapsulated it in a sapphire tube filled with argon gas; experiments have shown that this SFBG sensor, while maintaining high sensitivity, can operate stably at 1600 °C for over 20 h and is thus capable of adapting to the high-temperature and high-pressure environments of downhole conditions.

#### 2.1.2. Distributed Temperature Sensing

**Figure 2:**
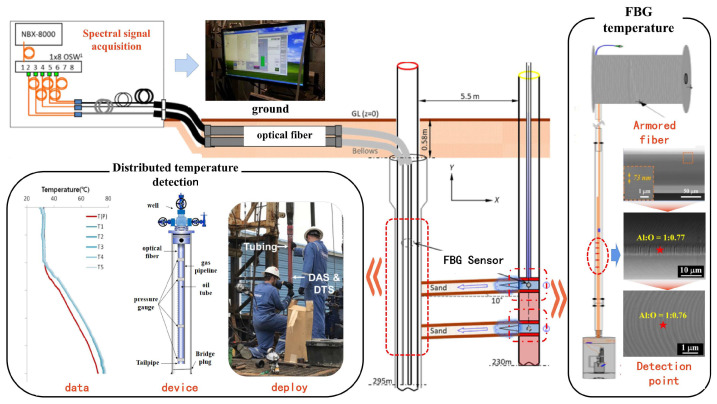
Downhole fiber optic temperature sensor [12,13,14,15,16].

Distributed temperature sensing (DTS) is a non-invasive, real-time optical-fiber sensing technology where the optical fiber serves dual functions of sensing and data transmission. It can be continuously deployed along the fiber and utilize the obtained distributed temperature data to predict and analyze other downhole physical parameters (Figure 2). The DTS temperature-measurement system consists of a set of interrogators that send, receive, and evaluate laser pulses. Based on the Brillouin scattering principle, it can calculate the frequency shift caused by fiber strain and temperature [14]:

(4) $$\Delta v_{B}=C_{v_{B}E}\Delta \varepsilon +C_{v_{B}T}\Delta T$$

In Equation (4), Δ*v_B_* represents the Brillouin frequency shift; Δ*ε* and Δ*T* denote the changes in fiber strain and temperature, respectively; C*_vBE_* and C*_vBT_* are the strain coefficient and temperature coefficient, respectively, and depend on the type of fiber and the operating wavelength. When the fiber is applied in a DTS system, if there is no strain in the fiber, the temperature can be directly derived from the frequency shift [14]:

(5) $$\Delta v_{B}=C_{v_{B}T}\Delta T$$

The Brillouin frequency shift, caused by changes in the optical phase, can respond with high sensitivity to temperature variations. Additionally, due to the distributed nature of the Brillouin frequency shift, it enables the detection of a wide temperature range over long distances. Therefore, research on distributed temperature sensing focuses on two main aspects: the need to reduce signal interference caused by fiber strain through compensation methods, thereby improving reliability, and the need to study sensor layouts to obtain rich distributed temperature data, which in turn provide solid data support for the evaluation of multiple downhole parameters. Utilizing DTS technology, a research team at Louisiana State University conducted temperature monitoring in a 1574-m deep well [15]. Temperature-measurement points were placed every 0.5 m along the wellbore, and the temperature data obtained were used to predict downhole pressure. The goodness-of-fit between the predicted and actual pressure values was greater than 0.95 at any measured depth. A research team at the University of Rennes, in France, used DTS temperature data to calculate the flow rates in different sections of the wellbore to quantify the flow rate of each productive fracture under various hydraulic conditions [16]. Distributed temperature-monitoring experiments in a 120-m-deep oilfield well showed that the flow-rate estimates based on DTS temperature data were highly consistent with the measured values. Researchers from The Hong Kong Polytechnic University have proposed a distributed monitoring method that can synchronously measure temperature and pressure [17]. This method utilizes the high sensitivity of frequency-scanning phase-sensitive photosensitive time-domain reflection to accurately demodulate the frequency shift of Rayleigh scattering and birefringence along polarization-maintaining fibers, which can effectively solve the problem of crosstalk between pressure and temperature. A research team from Taiyuan University of Technology [18] designed a wireless remote temperature-measurement system based on Raman distributed measurement, which they combined with a Raman distributed fiber temperature-sensing device and a 4 G data wireless transmission module to achieve wireless transmission of distributed temperature-sensing data. Compared with point-to-point wireless transmission, this method improves transmission speed and monitoring efficiency, and the difference between measured temperature and actual temperature is less than 1 °C.

### 2.2. Fiber-Optic Pressure Sensing

**Figure 3:**
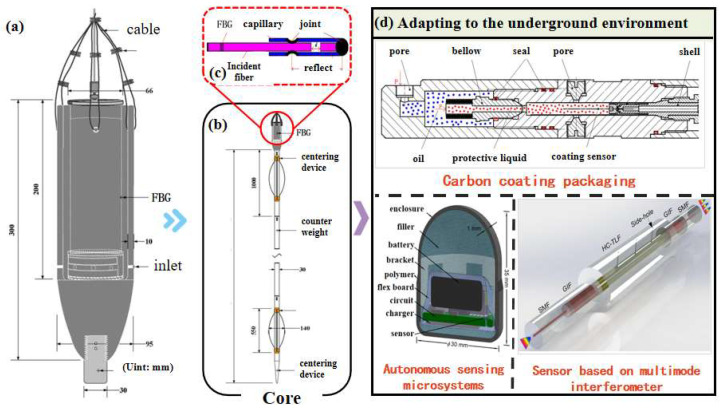
Downhole fiber optic pressure sensor. (**a**) Downhole pressure-sensor housing [12], (**b**) core of downhole pressure sensor [12], (**c**) typical structure of a pressure probe [22], (**d**) sensors with environmental adaptability [21,22,23].

Downhole pressure is a crucial parameter for evaluating the production status of oil and gas wells, with in situ monitoring primarily relying on fiber optic pressure sensing. Unlike fiber optic temperature sensing, which requires avoiding strain, fiber optic pressure sensing primarily detects strain induced by pressure (Figure 3). Taking classic Fabry–Perot interferometry as an example, when pressure *P* is applied to the sensor, the cavity length *d* decreases; moreover, the cavity length *d* is inversely proportional to the pressure *P*, and the ratio of the change in cavity length Δ*d* to the pressure *P* represents the pressure sensitivity of the sensor, which can be specifically expressed as follows [19]:

(6) $$\Delta d/P=\frac{L_{0}r_{0}^{2}}{E(r_{0}^{2}-r_{i}^{2})}(1-2\mu )$$

In Equation (6), *L*_0_ represents the gauge length; *r_i_* and *r*_0_ denote the inner and outer radii of the optical fiber, respectively; *E* is the elastic modulus of the fiber cladding material; and *μ* is the Poisson’s ratio of the material. Thus, the detection of changes in the optical signal makes it possible to determine the alteration in the length of the Fabry–Perot cavity, allowing for the inversion of downhole pressure information. However, for fiber optic pressure sensing, it is crucial to ensure that pressure is the sole source of fiber strain. In complex downhole environments, the impact of drilling fluids and the flow of other formation fluids can introduce signal interference to the pressure sensor [20]. Designing external protective mechanisms and detecting micro-cavities can effectively mitigate interference from other fluids. Additionally, thermal expansion of the fiber due to high downhole temperatures is a significant interference factor in fiber optic pressure sensing. Developing efficient methods to compensate for temperature drift is key to eliminating thermal-expansion interference, and integrating temperature sensing with fiber optic pressure sensing is an effective interference-suppression strategy. A research team in Shenzhen University has developed a non-diaphragm fiber optic gas pressure sensor based on a multimode interferometer [21] that splices a hollow-tube-lattice fiber to the outside of a multimode fiber and uses femtosecond laser technology to drill gas holes for gas flow, forming a pressure-detection chamber. This sensor exhibits high sensitivity of 8.1 nm/MPa and can withstand temperatures up to 400 °C, making it suitable for use in harsh deep-drilling conditions. China University of Geosciences has developed a carbon-coated bellows encapsulated fiber optic sensor for high-pressure and high-temperature downhole monitoring [22]. The sensor forms a Fabry–Perot (F–P) cavity, which acts as a pressure probe, in the gap between the incident and reflected fibers. When the probe is subjected to external pressure, the length of the F–P cavity changes, and demodulation the cavity-length change yields data that can be used to calculate the external pressure. This sensor has been successfully applied to pressure monitoring in a 1000-m deep well, with a pressure sensitivity of 1.48 nm/psi and a maximum pressure-measurement range of 10,298 psi at 150 °C. The University of Michigan has developed an autonomous downhole pressure microsystem integrated with sensors and electronic communication devices, suitable for multiphase flow coupling conditions [23]. The system’s data acquisition and processing are completed in situ downhole, with an outer layer of fluorocarbon elastomer encapsulation providing mechanical and chemical protection. This system has been successfully applied in a 1290-m deep oil well, recording pressure, temperature, and inertial data under conditions of 41.5 MPa and 85 °C. A research team at Northwestern University [24] has proposed an optical fiber pressure sensor based on a Fabry–Perot interferometer. Through the diaphragm structure at the optical fiber probe, the external pressure is converted into the deformation of the diaphragm, which causes the interference spectrum to shift. The sensor is used to monitor the pressure in 3500 m deep wells, showing good dynamic response ability and temperature resistance in the pressure range of 0–80 MPa.

### 2.3. Fiber-Optic Pipeline Safety Sensing

In the process of drilling engineering and oil and gas recovery, it is often necessary to lay a large number of pipelines to transport the recovered oil and gas, drilling fluid, and engineering waste. In in situ drilling and production of oil shale, some pipelines will be buried deep underground to withstand the complex environmental conditions of the formation. If there are safety hazards in the oil pipeline, they may directly affect the continuity and economic benefits associated with the pipeline and may even cause chain accidents in drilling operations. Fiber optic sensors have become a promising tool through which to monitor the integrity and safety of pipelines due to their small size, corrosion resistance, and other characteristics. A research team from North Dakota State University proposed a method for monitoring pipeline corrosion based on discrete-fiber Bragg gratings and distributed optical frequency domain reflectometry [25] and constructed a calculation model for corrosion pit depth and corrosion rate under impact load. The proposed method can maintain sufficient accuracy and stability at multiple time scales. A research team from Wuhan University has developed a method for locating leaks in natural gas pipelines based on a weak fiber Bragg grating array [26]. The reflectivity of weak fiber Bragg gratings is smaller than that of traditional Bragg gratings, and they have better adaptability in long-distance monitoring. The positioning error of this method is only 0.5 m. A research team from Aveiro University developed an accelerometer based on fiber Bragg gratings that can measure the natural frequency of pipelines [27]. The designed sensor successfully monitored the relationship between the degree of corrosion of pipelines and the natural frequency.

### 2.4. Downhole Gas–Liquid Sensing

During the drilling process, real-time measurement of the gas and liquid composition of the formation is a crucial method for assessing hydrocarbon production and ensuring drilling safety. As the drilling progresses deeper, harmful gases such as hydrogen sulfide from the formation may leak from the wellhead. Immediate identification and handling of these gases can effectively prevent drilling accidents. Real-time monitoring of the gas and liquid composition of the formation allows for the prompt and accurate identification of hydrocarbon production, as it makes it possible to determine the locations of hydrocarbon reservoirs. If samples from the drilling area are brought to the surface for testing, the accuracy of the detection relies on high-fidelity sampling techniques, and repeated drill string trips for sampling inevitably lead to delays in timeliness. Therefore, utilizing hydrocarbon sensing technology for downhole analysis of formation gas and liquid composition could significantly accelerate the progress of hydrocarbon exploration and production and enhance, drilling safety.

#### 2.4.1. Bionic Olfaction

Biomimetics, through long-term observation and study of the unique attributes of organisms, offers solutions to existing technological challenges [28]. The electronic nose (e-nose), composed of a series of gas sensors and corresponding pattern-recognition algorithms, mimics the olfactory systems of humans and mammals to identify the characteristics of gas and liquid components. It has been successfully applied in the recognition of gas and crude oil odors [29,30]. Universiti Teknologi PETRONAS in Malaysia has utilized the e-nose to specifically differentiate crude oil at temperatures ranging from 20 °C to 80 °C, achieving 100% recognition accuracy for crude oil [31]. A research team at The University of Cádiz has employed the e-nose to distinguish petroleum derivatives [32], achieving 97.7% accuracy in identifying different types of petroleum and varying degrees of volatilization without misclassifying volatilized petroleum as non-volatilized. Although the e-nose can differentiate various substances downhole, the electrochemical sensors commonly used in e-noses have poor resistance to high temperatures and pressures, making them unsuitable for direct installation near the drill bit. Future applications in downhole sensing while drilling are more focused on integration with fiber optic sensors.

#### 2.4.2. Crude Oil/Natural Gas Fiber Optic Sensing

Crude oil/natural gas fiber optic sensing boasts advantageous characteristics such as compact size, resistance to high temperatures and pressures, and immunity to electromagnetic interference, making it suitable for detection of crude oil in harsh downhole conditions. The Norwegian Geotechnical Institute has developed a fully distributed fiber optic sensor applicable for downhole oil and gas detection [33]. The sensor features a polyethylene elastomer coating on the fiber surface that expands upon absorbing hydrocarbons, thereby inducing strain in the fiber and enabling accurate determination of the crude oil’s stratigraphic location. A research team at the Malaysia University of Technology has fabricated a highly sensitive fiber optic probe for downhole detection of crude oil concentration. By removing part of the cladding and coating the sensing section with a zinc oxide/silver (ZnO/Ag) heterostructure layer, they altered the evanescent wave of the incident light within the coating. The transmission spectrum of the evanescent wave can be represented as follows [34]:

(7) $$\lambda_{m}=\frac{L\Delta n_{eff}}{m}$$

In the equation, *λ_m_* represents the *m*-th order displacement interference and Δ*n_eff_* is the difference between the refractive index of the coating and the refractive index of the fiber core. When the coating adsorbs crude oil molecules, its refractive index will change, and the m-th order shift interference can be expressed as follows [34]:

(8) $$\Delta \lambda_{m}=\frac{(\Delta n_{eff}+\Delta n)L}{m}-\frac{\Delta n_{eff}L}{m}=\frac{\Delta nL}{m}$$

The concentration of crude oil can be determined by the wavelength shift and intensity changes in infrared spectroscopy. Researchers at the Krakow University of Technology in Poland utilized a fiber optic Raman probe to conduct in situ measurements of natural gas composition in a gas well 271 m deep [35]. The signals collected by the probe were transmitted via optical fiber to a spectrometer, which converted them into the molar composition of the natural gas. This sensor is capable of monitoring the content of methane, nitrogen, and heavy hydrocarbons in the downhole natural gas. Integrating the light source into the Raman probe can shorten the optical path, enhance the robustness of the sensor, and reduce the impact of downhole noise on the detection results.

### 2.5. Engineering Drilling Parameters

**Figure 4:**
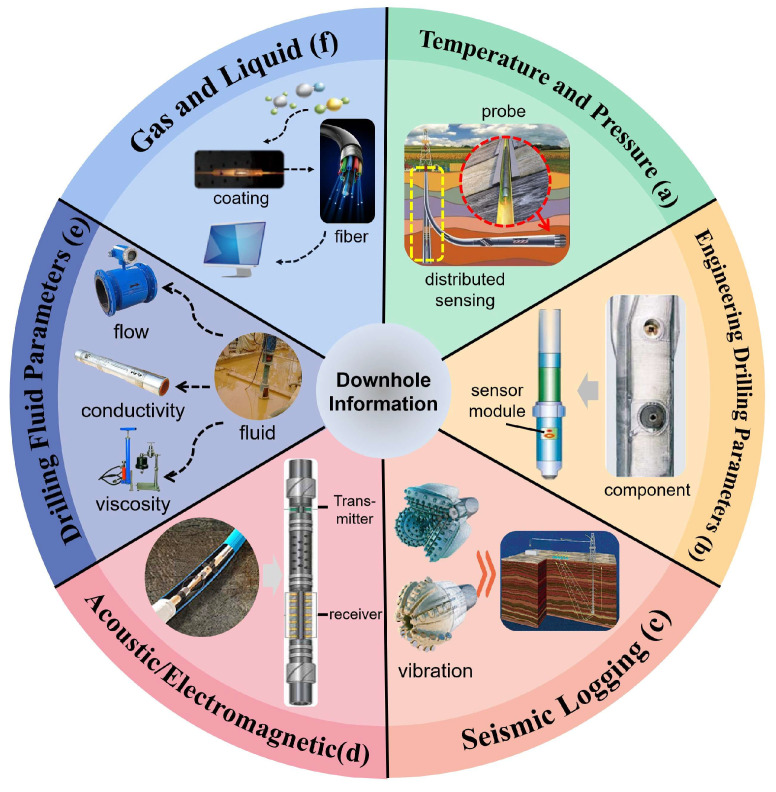
Method for underground information collection (**a**) Underground temperature and pressure sensing [12], (**b**) Engineering drilling parameter sensing [37], (**c**) Seismic logging while drilling [46], (**d**) Acoustic/electromagnetic wave logging [42], (**e**) Drilling fluid parameters [38] (**f**) Underground gas–liquid sensing [34].

During the drilling process, engineering drilling parameters can reflect the working status of the drilling rig via data including the rate of penetration (RPM), rotary table torque (TRQ), standpipe pressure (SPP), strokes per minute (SPM), weight on bit, and hook load (Figure 4) [36]. Engineering drilling parameter sensing can be divided into surface monitoring and downhole monitoring. The rate of penetration can be detected by installing a linear-displacement sensor on the drill pipe to measure the linear displacement of the drill pipe per unit time. The sensor is generally installed on the piston rod of the cylinder connected to the chuck or on the upper beam. The rotary table torque is obtained by measuring the tension of the rotary table chain. The torque sensor is fixed near the drive chain box of the rotary table, and the tension of the rotary table chain is transmitted to the hydraulic transmitter through hydraulic lines, converting the hydraulic signal into an electrical signal for collection. Monitoring of weight on bit can be achieved through in situ monitoring by installing a measurement sub above the drill bit [37]. The sub integrates a generator module, sensor module, data-acquisition module, and pulse-generation module. First, the sensor module collects voltage signals of various parameters, then the data-conversion module converts the voltage into binary data according to encoding rules, generating a set of square wave signals that are input into the pulse-generation module and transmitted back to the surface via pulse communication. Engineering drilling parameters are crucial for judging the drilling status, guiding operators to make preliminary judgments on the encountered formations, and improving the accuracy of signal acquisition to provide stronger information support for the analysis of drilling status. At the same time, as the drilling depth continues to increase, higher equipment reliability is required. Each module needs to maintain high sensitivity and precision in signal acquisition and stable data-transmission capabilities under high temperatures, high pressures, and the impact of drilling fluid.

### 2.6. Drilling Fluid Parameters

During the drilling mud-circulation process, the mud carries cuttings generated from the drilling zone, as well as liquids and gases from the formation, back to the surface. Therefore, real-time monitoring of various mud parameters can provide dynamic information indicating the drilling status. The main parameters of drilling mud include mud conductivity, mud viscosity, mud flow rate (MFO), mud flow velocity, and mud pit volume (MPV) [38]. Since the mud is detected on the surface and detection is not constrained by spatial conditions, only the sensitivity and resolution of the detection need to be considered. The mud flow information, such as flow rate and velocity, has good real-time characteristics and can be quickly detected using a drilling fluid flowmeter to indicate whether the formation pressure and wellbore pressure are balanced. However, the physical properties of mud, such as conductivity and viscosity, exhibit hysteresis and can be detected only after the mud has flowed from the drilled formation to the surface. Currently, in engineering practice, the physical properties of mud are mainly detected using resistance sensors, capacitance sensors, and viscometers [39,40,41]. It is important to emphasize that the flow characteristics of mud allow only for indirect inference of the properties of formation fluids and lithology. Synchronous collection of multi-source signals and integration of mud parameters with other downhole real-time drilling information can compensate for the hysteresis of mud parameters, enabling a more comprehensive understanding of the downhole drilling status.

### 2.7. Acoustic/Electromagnetic Wave Sensing

#### 2.7.1. Sonic Logging

Sonic logging while drilling evaluates formations and identifies fractures by determining the formation’s compressional and shear wave velocities, thereby identifying favorable reservoirs [42]. Sonic logging tools have been widely applied in the field of LWD, with core components including acoustic transmitters, acoustic receivers, and acoustic isolators. During the drilling process, the sonic logging tool is mounted on the drill string and rotates with it to achieve omnidirectional detection. Early sonic logging tools were equipped with only a single-directional acoustic transmitter that was capable of acquiring formation information within a narrow sector, resulting in low signal resolution. With technological advancements, it is now possible to achieve high-resolution LWD measurements across 16 directions [43]. In the future, the development will trend towards full-directional detection to meet the high-resolution requirements of LWD and downhole monitoring. Of course, full-directional detection implies the need for more transmitters and receivers, which will occupy more space. Therefore, sonic logging tools must also evolve towards miniaturization to accommodate the constraints of narrow wellbore spaces.

#### 2.7.2. Electromagnetic Wave Logging

Electromagnetic wave logging identifies lithological interfaces and determines tool orientation by measuring the attenuation of electromagnetic waves between coils, serving as a critical technology for real-time geological steering while drilling [44]. The core component of electromagnetic wave logging is the coil array, which is composed of transmitting and receiving coils. Conventional electromagnetic wave logging detects only the phase difference of electromagnetic waves and lacks capability for azimuth detection. Subsequent developments in electromagnetic wave logging have evolved to provide omnidirectional detection, achieved through axial, radial, and tilted configurations of the coil array. The detection signals have expanded from single-phase difference detection to simultaneous measurement of amplitude ratio and phase difference. The resolution of electromagnetic wave signals improves as the coil spacing decreases [45], while signal attenuation becomes more severe with increasing distance from the source. Therefore, the detection system should be positioned near the drill bit.

### 2.8. Seismic Logging

Drill-bit seismic while drilling (SWD) technology utilizes the vibrations generated by the drill bit impacting the formation during drilling as a seismic source for formation analysis and detection of the drill bit position [46]. The SWD logging system consists of surface geophones and a pilot sensor near the drill bit. The pilot signal from the pilot sensor is crosscorrelated with the surface signal to reduce surface-noise interference and minimize signal delay. However, the system is still limited by drill string transmission losses and borehole-noise interference, resulting in a relatively low signal-to-noise ratio for the overall detection system. The logging method using the drill bit’s vibration as a seismic source currently has certain limitations [47]: despite the use of noise-reduction techniques, a significant amount of borehole noise still permeates the logging data along the time axis. Additionally, during the drilling process, the drill bit inevitably experiences wear, causing changes in its vibration wavefield and leading to distortion in the logging data.

## 3. Intelligent Downhole Data Processing Technology

**Figure 5:**
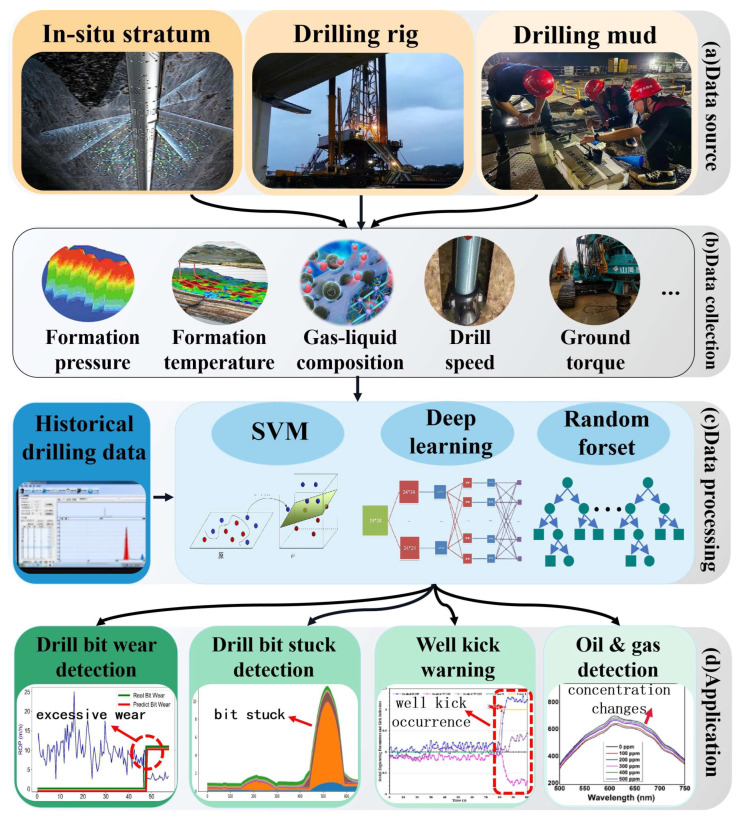
Process of real-time perception of drilling and production status: (**a**) data sources [36,38], (**b**) data acquisition [7], (**c**) algorithm processing [48,49,50], (**d**) application examples [51,53,56,61].

Through situ collection of downhole physical parameters, effective downhole information can be promptly obtained. However, drilling sites often simultaneously collect multi-source drilling data, including downhole temperature, pressure, bit rotation speed, torque, weight on bit, and drilling speed. Relying solely on the experience of surface operators to assess drilling conditions can lead to misjudgments of abnormal conditions and delayed responses to anomalies. Intelligent monitoring technology for downhole drilling condition recognition utilizes machine learning and other intelligent algorithms to analyze drilling data, establishing models for drilling-state identification and reservoir prediction. This technology can alert operators to the need to handle anomalies in the early stages of abnormal drilling conditions or provide early predictions of potentially favorable reservoirs to guide drilling direction, effectively preventing accidents, reducing operational costs, and enhancing drilling efficiency (Figure 5).

### 3.1. Typical Intelligent Algorithms

Intelligent algorithms are capable of simulating human learning behaviors by utilizing various nonlinear mapping methods to extract key feature information from complex high-dimensional data and determine class boundaries or intrinsic trends in feature changes, thereby making advanced decisions that traditional human experience cannot achieve. According to task attributes, intelligent algorithms can be divided into pattern classification and regression analysis; according to learning rules, they can be categorized into supervised, semi-supervised, unsupervised, and reinforcement learning.

In the field of intelligent monitoring, based on the task requirements of actual drilling scenarios, they mainly include two categories: drilling-state recognition and prediction of oil and gas production, which are essentially pattern classification and regression analysis, respectively. As a representative of kernel methods, support vector machine (SVM) [48] is a typical classification model. For linearly separable data, SVM classifies the data by finding the line that maximizes the distance between adjacent points and independent points; for linearly inseparable data, it uses specific kernel functions to map the data from low-dimensional space to high-dimensional space, transforming it from low-dimensional inseparable to high-dimensional separable, thereby completing the classification of data. It is widely used in areas such as formation identification and stuck-pipe monitoring. As an important application branch of SVM, support vector regression minimizes the distance to the farthest sample points from the hyperplane using kernel functions and has been applied in leak-risk assessment for drilling and prediction of oil and gas production. Random forest [49], a classification and regression algorithm based on decision tree ensembles, consists of multiple decision trees that are independent of each other. When new samples are input, each decision tree independently provides its own classification or regression result, and the final output of the random forest is obtained by merging the prediction results of all decision trees through the bagging mechanism. Implementing a typical ensemble learning strategy, random forests can effectively solve the overfitting problem of single decision trees with fast classification speed, simple models, and high accuracy, characteristics that make them suitable for pattern classification of multidimensional correlated drilling data with redundancy. Deep neural networks [50], an emerging intelligent algorithm in the field of machine learning, differ from traditional manual feature engineering or statistical learning-based machine learning methods. Being “data-driven” is the most significant characteristic of deep neural networks. Conventional deep neural networks contain multiple hidden layers, each with a large number of neurons. When a large amount of labeled data is input into the neural network for training, neurons continuously update their weights and biases based on errors, autonomously learning classification or regression rules from the training data, thereby achieving end-to-end pattern classification or regression analysis. In recent years, deep neural networks represented by convolutional neural networks, long short-term memory neural networks, and generative adversarial networks have garnered significant attention in the field of drilling engineering. Convolutional Neural Networks are primarily used to process geological or formation images derived from multi-source sensor inversions; long short-term memory neural networks are mainly applied in the analysis of sensor time-series signals; and generative adversarial networks are commonly used for data augmentation under limited data conditions. Additionally, bio-inspired intelligent optimization algorithms such as particle swarm optimization, simulated annealing, and genetic algorithms have also been applied in multi-objective optimization of drilling parameters, feature selection of drilling parameters, and parameter optimization of pattern-classification algorithms.

By integrating intelligent algorithms with in situ detection methods based on micro-sensors, smart monitoring technology can accurately perceive drilling conditions, enabling intelligent analysis of multi-modal data such as drilling operations and oil and gas production. This facilitates safe drilling and enhances drilling efficiency.

### 3.2. Real-Time Monitoring of Drill Bit Wear

During the drilling process, as the depth increases and the encountered formations continuously change, the drill bit inevitably experiences wear and requires multiple replacements to ensure drilling efficiency. It is essential to carefully evaluate the timing of bit replacement to avoid unnecessary tripping, which can lead to additional drilling costs and affect the drilling progress. Real-time monitoring of drill bit wear is essentially a combination of pattern recognition and regression analysis. The pattern-recognition part primarily uses sensor data to identify whether the drill bit is worn, while the regression analysis part establishes the distribution of wear-recognition results and sets an appropriate wear-alarm threshold based on prior knowledge.

Combining probabilistic neural networks (PNN) and Bayes’ theorem, researchers at São Paulo State University in Brazil have designed a real-time evaluation model for drill bit wear. This model uses surface torque data collected from early drilling operations to train the neural network, resulting in a preliminary wear-state-recognition model for drill bits. The output of the neural network is obtained through iterations of its nodes [51]:

(9) $$\varphi_{ij}(x)=\frac{1}{\sqrt{2\pi }\sigma }\exp\left[-\frac{{\left(x-x_{ij}\right)}^{2}}{2\sigma^{2}}\right]$$

In Equation (9), *φ_ij_* represents the estimated value of wear degree; *σ* is the smoothing factor; *x* and *x_ij_* denote the test sample and training sample, respectively. After the wear-prediction results for drill bits have been obtained, the reliability of the model’s output is verified using Bayes’ theorem, with the results ultimately determining whether the drill bit needs to be replaced. This model can provide a prompt for drill bit replacement within a 30-min window of the optimal replacement time, significantly improving the efficiency of drill bit wear monitoring. A research team from Harbin Institute of Technology has proposed a multi-condition drill bit wear prediction model based on a variable-condition intervention neural network [52]. This method uses drill bit wear images as the data source and extracts features insensitive to time-invariant conditions through a domain adversarial mechanism, thereby improving the model’s accuracy under variable working conditions. The accuracy and stability of this model are superior to existing statistical and machine learning models.

The variation in drill bit wear is a dynamic process significantly influenced by geological formations. The responsiveness of the drill bit wear-prediction model heavily depends on the resolution of drilling data, which exhibits temporal correlations. Therefore, employing high-sensitivity, high-resolution data-acquisition methods combined with efficient time-series processing algorithms is crucial for enhancing the monitoring capability of drill bit wear.

### 3.3. Drilling Stuck Real-Time Monitoring

Stuck pipe is a severe drilling accident that can have catastrophic impacts on the drilling process, including time delays and damage to drilling equipment. Therefore, the goal of real-time stuck-pipe monitoring is to utilize multi-source drilling sensor data and intelligent analysis algorithms to predict the likelihood of stuck pipe, providing timely warnings before it occurs, assisting engineers in resolving the issue, and averting the adverse consequences of stuck pipe.

Researchers at King Fahd University of Petroleum and Minerals developed an intelligent early warning system [53] that uses random forests, artificial neural networks, and function networks to establish models for stuck-pipe prediction based on data collected from drilling sites, including flow rate, hoisting load, drilling speed, rotation speed, standpipe pressure, and weight on bit. This system has been successfully applied in a carbonate gas reservoir-drilling operation at 1524 m deep, where it detected abnormal conditions and issued stuck-pipe warnings up to 9 h in advance. Researchers in The Jianghan Machinery Research Institute of China National Petroleum Corporation proposed a method fpr stuck-pipe prediction combining linear regression, artificial neural networks, and fuzzy inference [54]. This method obtains a friction coefficient representing the trend in stuck pipe through linear regression of drilling parameters, trains an artificial neural network to identify stuck pipe incidents, and finally uses fuzzy inference to establish a comprehensive stuck-pipe index to obtain the probability of stuck pipe. The proposed method was validated using stuck-pipe data from the Tarim Oilfield, and experimental results showed that the model achieved a 98% accuracy rate in stuck-pipe prediction, with only a 1% false alarm rate. Researchers at China University of mining and technology [55] proposed a real-time method to predict sticking based on long-term and short-term memory neural network. The main input characteristics are torque, rotating speed, weight on bit, and rate of penetration parameters; these are combined with the bit-adhesion characteristics when sticking occurs. The model can predict the occurrence of sticking accidents four seconds in advance.

Intelligent algorithms for stuck-pipe accident prediction should have low computational complexity and efficient feature extraction and reasoning capabilities, enabling them to extract intrinsic characteristics representing stuck-pipe risk from large amounts of drilling data. Although current models for stuck-pipe prediction can effectively predict specific stuck-pipe conditions, most models are built for specific drilling conditions and have poor generalizability. In future research, methods such as transfer learning could be introduced to existing models to enable the reuse of models for stuck-pipe prediction, or big data-based analysis methods could be used to identify common features of stuck pipe across different drilling equipment, thereby establishing more generalizable models for stuck-pipe prediction.

### 3.4. Real-Time Monitoring for Well Kick

When the formation pressure exceeds the wellbore pressure, the pressure differential can cause formation fluids to flow into the wellbore, leading to a well kick. If timely measures are not taken, a blowout accident may occur. Therefore, it is essential to provide early warnings before a well kick happens or issue alerts during the early stages of a well kick to prevent harm to drilling site personnel. Key indicators such as drilling fluid flow rate and pressure are critical for detecting whether formation fluids are entering the wellbore. Thus, the use of flow and pressure sensors to collect wellbore and formation pressure data, combined with intelligent algorithms for multi-parameter integrated analysis, is crucial for well kick early warning. Intelligent algorithms capture the changing patterns of key features throughout the well-kick process by constructing feature spaces for different operating conditions, enabling timely warnings before a well kick occurs.

A research team at Memorial University of Newfoundland in Canada trained a long short-term memory neural network to detect changes in the slope of normalized penetration rate and the peak reduction in riser pressure, providing early warnings during the initial stages of a well kick [56]. Applications in four offshore deviated wells demonstrated that this method could issue alerts within five minutes of a well kick. China University of Petroleum proposed a method for early warning and identification of well kicks based on LSTM neural networks [57], using riser pressure, drilling fluid inlet and outlet flow rates, and other parameters as model inputs. The method categorizes well-kick risks into four levels, assessing the danger and stage of a well kick. In a deepwater drilling operation in the South China Sea at a depth of 4450 m, the model achieved an identification accuracy of 87% and was able to detect well kicks earlier than traditional mud flow-detection methods, prompting operators to take different measures for well-kick management. China University of Geosciences employed clustering analysis to extract time-series features of drilling parameters related to well-kick risks and input them into a Bayesian classifier to determine the occurrence of a well kick [58]. This method, applied in a 2800-m-deep drilling operation, achieved an identification accuracy of 95.95%, enabling low rates of false alarm and missed detection for identification and prediction of well kicks.

In addition to flow rate and pressure, other parameters such as mud conductivity and acoustic echo intensity may also exhibit abrupt changes before a well kick. Therefore, it is advisable to integrate flow rate, pressure, and other drilling parameters at the feature or decision level to achieve complementary information and improve the accuracy of well-kick prediction. When predicting well kicks, it is also necessary to consider sudden formation changes that cause pressure differential jumps, taking into account factors such as drilling speed, pressure differential fluctuations, and rotational speed variations to reduce rates of false alarms.

### 3.5. Identification of Favorable Reservoirs and Petroleum Production

Formation evaluation and reservoir monitoring are crucial for optimizing reservoir production, predicting reservoir reserves, and assessing hydrocarbon output. During the drilling process, real-time analysis of formation conditions and hydrocarbon output using intelligent algorithms can guide surface operators to promptly correct wellbore trajectories, adjust drilling parameters, and maintain the drill bit in the optimal reservoir position, thereby enhancing rates of hydrocarbon recovery.

A research team at Vietnam’s Duy Tan University has proposed a method for monitoring oil output using neural networks to analyze gamma radiation data for formation fluids [59]. By inputting photon-attenuation signals from radioactive elements in formation fluids collected by a dual-energy gamma-source-detection system into an artificial neural network, the method identifies the categories and contents of ethylene glycol, crude oil, light oil, and gasoline with an error rate less than 3%. Researchers at the Australian College of Kuwait have developed a deep convolutional neural network-driven method for identifying favorable reservoirs based on formation pressure transient signals [60]. When historical drilling-pressure time signals are input into a deep convolutional neural network for training, the established model can accurately distinguish different reservoirs based on real-time transient changes in formation pressure monitored at the drilling site, achieving an overall classification accuracy of 98.36%. Moreover, the model exhibits strong robustness, maintaining high recognition rates under noisy and signal-missing conditions. Researchers at the University of Stavanger in Norway have designed a model to predict oil and gas production based on an autoregressive recurrent neural network [61]. This model uses time-series data of formation pressure and drilling-fluid flow rate as inputs to assess current oil-production rates and predict future output. The model was field-tested in the Midland Oilfield in the United States, where its short-term production rate predictions closely matched actual production rates over a 48-month drilling period, significantly outperforming manual prediction methods. The Polish National Renewable Energy Research Institute [62] combined neural network technology with seismic logging data to achieve preliminary qualitative and spatial characterization of carbonate reservoirs and identified the areas most likely to form oil and gas accumulation and areas likely to show enhanced reservoir characteristics. To achieve in situ specific identification of downhole hydrocarbon gas components, a research team at Jilin University has proposed a miniaturized bionic electronic-nose system for downhole oil and gas detection [63]. The system enhances gas signals through bionic designs such as a human nasal-turbinate structure, shark-skin V-groove surface, and skin-surface flow field distribution in the electronic nasal chamber. It uses a support vector machine to identify hydrocarbon gas components, with its compact and miniaturized structure adapting to the confined downhole space, achieving an overall recognition accuracy of over 87%. For favorable reservoir and crude oil identification, the strong correlation and complementarity among oil, gas, and vibration sensing units deployed at different drilling positions necessitate information decoupling and feature fusion of various types of sensing signals to improve the recognition accuracy of intelligent algorithms.

## 4. Application Prospects of Intelligent Monitoring Technology

With the continuous growth in energy demand and the advancement of extraction technologies, the focus of resource extraction has expanded beyond merely exploring and developing new oil and gas fields. The vast potential resources hidden in the deep earth, the deep sea, and deep space are gradually gaining attention [64,65,66]. Additionally, for major scientific exploration projects related to the origins of life and planetary evolution, the deep earth, the deep sea, and deep space hold significant scientific value, providing crucial support for understanding the Earth’s internal structure and the history of planetary evolution. The advantages of in situ multisource data collection, efficient information transmission, and intelligent data analysis offered by downhole sensing and intelligent monitoring technologies make them powerful tools for future resource exploration and scientific endeavors.

### 4.1. Deep and Ultra-Deep Scientific Exploration and Resource Prospecting

The mineral-forming space within the earth extends from the surface down to a depth of 10,000 m. The resources and energy stored in the earth’s deep interior form the material and energy foundation that sustains all life. Conducting scientific exploration and resource prospecting in deep and ultra-deep layers not only addresses significant fundamental theoretical issues in geoscience but also ensures the security of energy resources and supports the socio-economic development of our country.

Recently, researchers have commenced drilling a new 10,000-m scientific exploration well. This project faces a series of challenges, including ultra-deep drilling depths, extremely difficult drilling strata, and multiple pressure coefficients. The geological parameters in deep and ultra-deep layers are complex, and the environment is harsh. When utilizing intelligent monitoring systems for deep and ultra-deep scientific exploration, it is necessary to design sensing and detection modules that can withstand high temperatures, high pressures, and high humidity. These modules integrate multiple sensing units to achieve synchronous multi-source signal acquisition, thereby constructing a micro-intelligent monitoring system to overcome the challenges of deep drilling. By integrating high-performance mechanical and speed sensing units, the drilling speed and drill torque can be monitored to adjust drilling parameters and prevent jamming and breakage. Additionally, combining logging-while-drilling methods to obtain information about the encountered strata and drilling direction, intelligent algorithms can be used for real-time geological steering, adjusting the drilling direction to prevent deviation. High-precision pressure sensing units are integrated for real-time pressure measurement, enabling intelligent risk assessment to prevent blowouts and ensure the safety of deep drilling.

As an advanced shale-oil extraction technology, in situ conversion of oil shale involves heating underground oil-shale formations to pyrolyze kerogen into shale oil and gas [67]. Compared to surface retorting methods, this approach offers lower pollution, reduced water consumption, and higher product quality [68,69]. During the in situ conversion process, the pyrolysis products vary depending on the stage of kerogen pyrolysis [70]. Current methods for assessing the degree of oil-shale pyrolysis, such as vitrinite reflectance and rock-eval pyrolysis (RE), have certain measurement limitations and cannot reflect the pyrolysis stage in real-time, potentially leading to incomplete or excessive pyrolysis and thus resulting in reduced oil and gas output and energy waste [71]. The in situ and rapid detection capabilities of intelligent monitoring technology provide the possibility for precise monitoring of the degree of oil-shale pyrolysis. In future shale-oil engineering projects, intelligent monitoring probes integrating multiple sensors for oil, gas, temperature, pressure, and spectroscopy can be deployed at various points in heating and production wells to comprehensively perceive the pyrolysis process and products from multiple physical perspectives. The embedded intelligent algorithms in these probes can quickly identify pyrolysis products, establish spatiotemporal distribution maps of temperature, pressure, and oil and gas products in the pyrolysis zone and infer the pyrolysis stage and degree based on product types and changes in oil and gas content. The intelligent monitoring-based method for identifying oil-shale pyrolysis is expected to become a universal approach for monitoring the pyrolysis stages of various types of shale under different geological conditions, optimizing shale-oil production processes, improving recovery rates for oil and gas, and reducing production costs [72].

### 4.2. In Situ Exploration of Polar and Deep-Sea Regions

**Figure 6:**
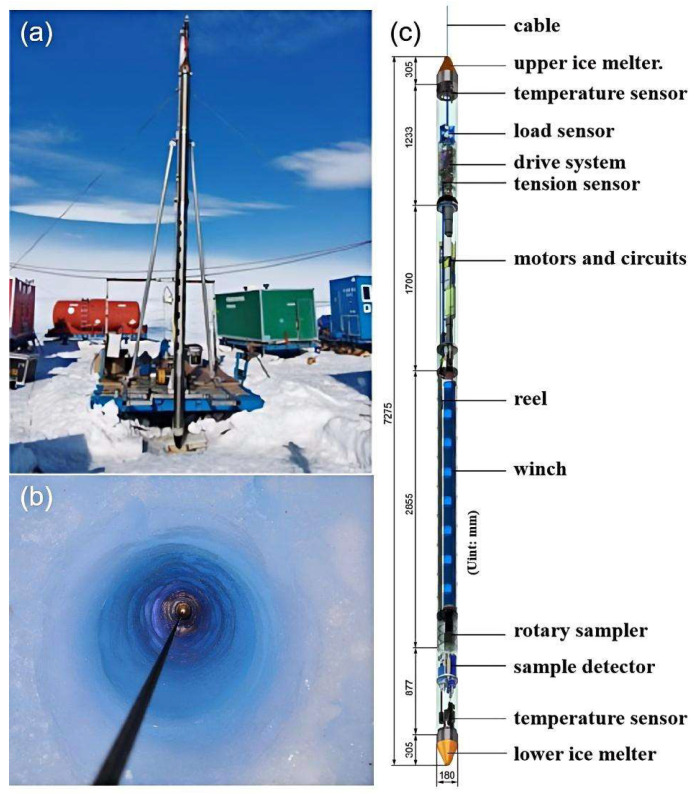
Jilin University recyclable autonomous sonde [73] (**a**) RECAS testing site at Zhongshan Station in Antarctica, China, (**b**) RECAS deployment under polar ice caps, (**c**) RECAS working 3D model.

Conducting in situ exploration of polar deep seas to study the biological gene pools contained in the polar regions and the oil and gas mineral resources buried in the deep sea holds significant scientific and strategic value for revealing global climate change, the evolution of life on earth, and the development of new energy sources. The physicochemical properties of subglacial lake water in polar regions are of great importance for studying ancient environments and climates, exploring polar life forms, and developing polar resources. Due to differences in overlying ice thickness and temperature distribution, there are stringent requirements for scientific drilling methods and non-destructive water sampling in polar subglacial lakes. The retrievable autonomous sounding instrument (RECAS) developed by the Polar Research Center of Jilin University can sample and analyze subglacial water while remaining isolated from the surface [73]. This device penetrated a 200.3-m-thick polar ice sheet, sampled meltwater, and measured its pressure, temperature, pH, and conductivity (Figure 6). During the thermal drilling process in subglacial lakes, it is essential to avoid repeated drilling to minimize contamination from the external environment and to measure as many in situ parameters as possible, including environmental and physicochemical parameters of the meltwater, in a single drilling operation. Therefore, it is advisable to consider adding a scientific payload module to the existing sounding instrument to install an intelligent in situ monitoring system integrated with physicochemical analysis functions. This system would enable continuous monitoring of the drilling process and real-time analysis of samples, creating a vertical profile of physicochemical parameters throughout the ice sheet and constructing a model that links physicochemical parameters, environmental factors, and microbial communities. This would help uncover the patterns of changes in environmental factors and their impact on subglacial lake ecosystems, revealing the life forms in subglacial lakes and inferring the early evolution of life on Earth and ancient climate processes. It is important to note that the high-humidity and low-temperature environment of subglacial lakes can interfere with sensors. When constructing an intelligent in situ monitoring system, it is necessary to establish a decoupling model, such as one based on neural networks, to address the coupled interference of temperature and humidity. Based on the decoupling results, corresponding automatic control and adjustment structures should be designed for humidity and temperature interference, ensuring that the intelligent in situ monitoring system operates in an ideal environment and maintains the accuracy of monitoring and analysis results.

Beyond the in situ exploration of subglacial lakes in polar regions, the deep ocean harbors vast untapped marine biological resources, seabed chemical resources, deep-sea mineral resources, marine spatial resources, and deep-sea energy resources [74]. Through the integration of various types of sensors, including electrical, chemical, electrochemical, and optical sensors, into intelligent monitoring systems, it is possible to detect and analyze various physical and chemical properties of deep-sea water and seabed materials, such as light intensity, dissolved gases, and compounds. This provides hydrological information support for deep-sea resource exploration [75]. In subsequent research efforts, on one hand, we can start with the hardware composition of intelligent monitoring systems, increasing the types and numbers of sensors to enhance the diversity of detectable substances and the richness of sensing information. On the other hand, attention should also be paid to the application of algorithms. We can attempt to fuse information from various sensors based on strategies such as feature-level and decision-level fusion, using intelligent algorithms to extract key features from complex, high-dimensional, and massive data. This will improve the representation performance of intelligent monitoring systems, reduce the need to blindly deploy detectors, and thereby achieve efficient deep-sea resource exploration.

### 4.3. Extraterrestrial Planetary Sampling

**Figure 7:**
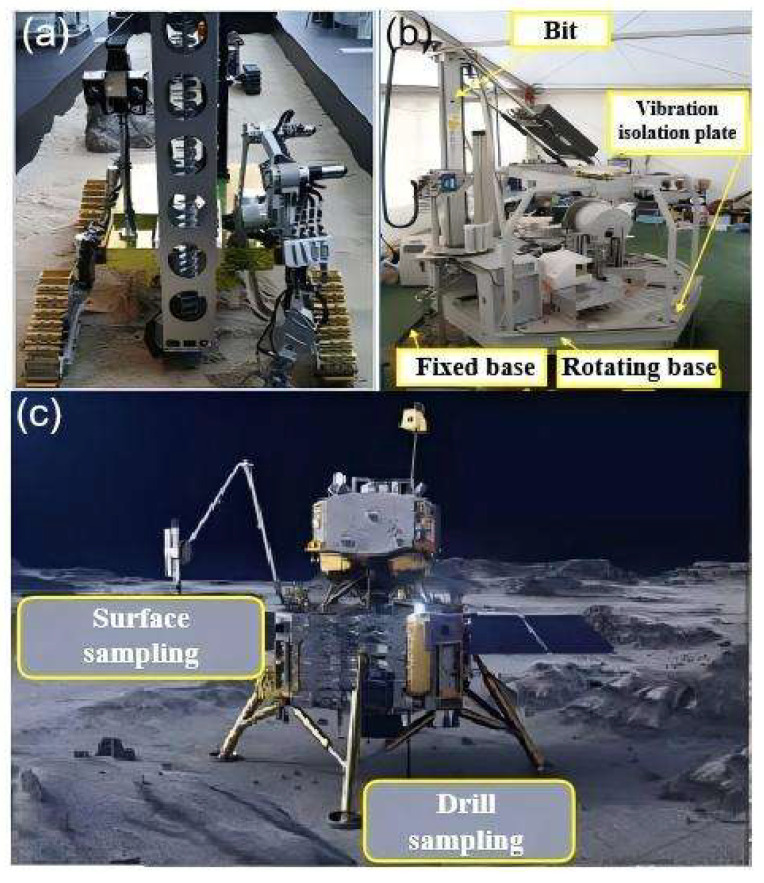
Unmanned system for sampling/returning extraterrestrial objects: (**a**) weathering layer sampler—lunar polar-ice-sampling rover [77]; (**b**) MARTE robot system [78]; (**c**) Chang’e-5 and sampler [79].

Extraterrestrial bodies possess abundant mineral resources and clean energy. However, due to the current limitations in planetary-exploration technology, there are constraints related to ground-based technology, planetary environments, and transmission distances (Figure 7) [76]. Human research on extraterrestrial sample-return missions has primarily focused on the Moon and Mars. For lunar and Martian sampling missions, the Japan Aerospace Exploration Agency (JAXA) has developed an unmanned rover [77] to search for water ice in the polar regions of the Moon. The rover is equipped with a physicochemical analyzer to analyze the physical and chemical properties of collected samples. NASA has designed the MARTE robotic system for drilling and sampling Martian regolith [78]. This system, through its onboard sample-detection platform, can perform basic sample processing, characterization, and life detection. China’s independently designed Chang’e-5 lunar probe successfully landed in a preselected area on the near side of the Moon and returned 1731 g of lunar samples, marking China’s first unmanned lunar sample-return mission. Whether for the Moon or Mars, achieving rapid, in situ, and accurate intelligent analysis of extraterrestrial samples is of great significance for planning future payloads and enhancing the value of returned samples.

Currently, the systematic application of downhole sensing and intelligent monitoring technologies in extraterrestrial-exploration robots and sample-return vehicles has not been reported. The application of downhole sensing and intelligent monitoring to extraterrestrial sample collectors is feasible and offers the following advantages:

(1) Downhole sensing and intelligent monitoring technologies can obtain the most authentic first-hand data on extraterrestrial samples with minimal damage, preventing potential information loss from samples during long-distance interplanetary transportation.

(2) The “measure-while-drilling” approach can support the selection of high-value target samples, enabling scientific downlink payload planning.

(3) Downhole sensing and intelligent monitoring can meet the specific requirements of applications such as detection of extraterrestrial life.

Future downhole sensing and intelligent monitoring technologies for extraterrestrial sample return missions need to address the following three aspects:

(1) Due to the payload limitations of launch vehicles and the size constraints of landers, the intelligent monitoring system must be as miniaturized as possible. This can be achieved by leveraging key technologies such as microfluidics, MEMS processes, and embedded processors to develop on-chip intelligent monitoring sensors, meeting the sampler’s requirements for small size, low energy consumption, and integrated sensing, computing, and storage.

(2) The significant communication delay in extraterrestrial environments makes real-time effective control from ground control centers impossible. Therefore, the intelligent monitoring system must possess a high degree of autonomous decision-making capability to handle various unexpected situations and improve system reliability. Additionally, constrained by limited computational resources and energy supply, the analysis unit of the intelligent monitoring system should be “small yet precise,” enabling accurate and rapid intelligent analysis of samples with low consumption of computational resources, thereby supporting selective sample return.

(3) Extraterrestrial planets experience extreme temperature fluctuations and intense particle radiation. It is crucial to focus on temperature regulation and radiation protection for the intelligent monitoring system. Special thermal insulation and electromagnetic shielding materials should be used to isolate heat sources and provide electromagnetic protection. Based on this, temperature-regulation algorithms should be designed to achieve constant temperature control of the intelligent monitoring system, effectively coping with various complex environmental changes and ensuring system reliability.

Finally, it is particularly important to emphasize that due to their unique geographical locations, the deep earth, the deep sea, and deep space often lack the conditions for conducting on-site pre-trials. Extensive remote simulation tests are required before the formal implementation of related engineering or scientific exploration projects. However, constrained by the limitations of simulation conditions, it is impossible to exhaust all possible scenarios, especially under extreme conditions such as high temperatures, high pressures, and extreme depths. There is an urgent need for alternative solutions that allow for repeated testing at a lower cost. The emergence of digital twin technology [80] holds promise in overcoming this challenge. During the pre-experimental simulation process, by constructing a digital twin of the physical equipment, it is possible to simulate the equipment’s working conditions under extreme environments in the digital space, thereby obtaining a large amount of realistic data to facilitate iterative corrections to the design and equipment. The application of digital twins in simulated deep-earth, deep-sea, and deep-space conditions can to some extent overcome the difficulty associated with conducting on-site trials, providing effective support for evaluating equipment performance. Furthermore, alterations to the environmental parameters of the twin can quickly simulate the equipment’s working conditions in other usage environments, helping engineers comprehensively understand the equipment’s operational status under various extreme conditions. At the same time, during actual scientific exploration and resource prospecting processes, digital twin technology can utilize sensor arrays on physical equipment to obtain equipment information, update the digital twin in real time, and feed the twin’s subsequent behavior back to the physical entity. In the digital space, monitoring the twin’s status can allow the equipment’s operational parameters to be determined, helping users better understand the equipment’s condition. Additionally, relying on a vast library of historical simulation results, the digital space can simulate the equipment’s environment and predict potential geological formations encountered during drilling, thereby adjusting the drilling direction and optimizing the wellbore trajectory.

## 5. Conclusions

The accurate acquisition and intelligent processing of drilling information are two critical aspects of drilling operations, essential for precisely understanding drilling conditions and evaluating oil and gas production. Over the past few decades, significant advancements have been made in the methods of acquiring and processing drilling information. The progress in downhole sensing technology has, to some extent, overcome challenges such as difficult surface sampling, high latency, and poor accuracy, shifting information collection from the surface to downhole operations. This shift has matured over time, leading to the development of various downhole sensing methods tailored to different operational conditions. Building on this foundation, the processing of drilling information is rapidly advancing towards intelligence and systematization. Core technologies like machine learning have facilitated a transition from manual analysis to intelligent processing, significantly enhancing downhole sensing types, comprehensive utilization of sensing information, and monitoring performance.

As technology progresses, humanity is gradually gaining more comprehensive capabilities for deep-earth, deep-space, and deep-sea exploration. Downhole sensing and intelligent monitoring technologies must continuously improve their detection capabilities to meet the demands of exploration and enhance system performance to cope with increasingly complex geological conditions and harsh working environments. Trends in the future development of downhole sensing and intelligent monitoring technologies can be summarized into four categories:

(1) High-performance sensors: sensors with superior characteristics such as high sensitivity and low detection limits that can be used to accurately perceive physical parameters like formation temperature and pressure in real time, providing more reliable and sufficient data support for subsequent analysis.

(2) High-resolution multi-source signal synchronous acquisition systems: circuit systems that efficiently coordinate hardware and software resources used to achieve synchronous, high-resolution acquisition of multi-source sensor signals, enabling multi-angle and comprehensive perception of drilling parameters to effectively improve the accuracy of monitoring systems.

(3) High-reliability system components: higher-performance materials with protection mechanisms and error-correction functions to ensure that system components such as sensing, transmission, computation, and storage have excellent anti-interference performance and can operate normally in various complex environments.

(4) Miniaturization and intelligent monitoring systems: downhole sensing and intelligent monitoring systems that have developed towards miniaturization, ensuring steady improvement in the information sensed while meeting the volume constraints of narrow wellbore spaces. Additionally, as the amount of information sensed by sensors increases, the system must also possess stronger information-processing capabilities, enabling downhole sensing and intelligent monitoring systems to handle increasingly complex scientific-exploration and energy-exploration tasks, thereby safeguarding national energy security.

## Data Availability

Not applicable due to it is a review.
